# Dengue Virus-Infected Dendritic Cells, but Not Monocytes, Activate Natural Killer Cells through a Contact-Dependent Mechanism Involving Adhesion Molecules

**DOI:** 10.1128/mBio.00741-17

**Published:** 2017-08-01

**Authors:** Vivian Vasconcelos Costa, Weijian Ye, Qingfeng Chen, Mauro Martins Teixeira, Peter Preiser, Eng Eong Ooi, Jianzhu Chen

**Affiliations:** a Interdisciplinary Research Group in Infectious Diseases, Singapore–MIT Alliance for Research and Technology, Singapore, Republic of Singapore; b School of Biological Sciences, Nanyang Technological University of Singapore, Singapore, Republic of Singapore; c Humanized Mouse Unit, Institute of Molecular and Cell Biology, Agency of Science, Technology and Research, Singapore, Republic of Singapore; d Institute of Biological Sciences, Federal University of Minas Gerais, Belo Horizonte, Minas Gerais, Brazil; e Duke-National University of Singapore Medical School, Singapore, Republic of Singapore; f Koch institute for Integrative Cancer Research and Department of Biology, Massachusetts Institute of Technology, Cambridge, Massachusetts, USA; Icahn School of Medicine at Mount Sinai

**Keywords:** Dengue virus, adhesion molecules, dendritic cells, humanized mice, natural killer cells

## Abstract

Natural killer (NK) cells play a protective role against dengue virus (DENV) infection, but the cellular and molecular mechanisms are not fully understood. Using an optimized humanized mouse model, we show that human NK cells, through the secretion of gamma interferon (IFN-γ), are critical in the early defense against DENV infection. Depletion of NK cells or neutralization of IFN-γ leads to increased viremia and more severe thrombocytopenia and liver damage in humanized mice. *In vitro* studies using autologous human NK cells show that DENV-infected monocyte-derived dendritic cells (MDDCs), but not monocytes, activate NK cells in a contact-dependent manner, resulting in upregulation of CD69 and CD25 and secretion of IFN-γ. Blocking adhesion molecules (LFA-1, DNAM-1, CD2, and 2β4) on NK cells abolishes NK cell activation, IFN-γ secretion, and the control of DENV replication. NK cells activated by infected MDDCs also inhibit DENV infection in monocytes. These findings show the essential role of human NK cells in protection against acute DENV infection *in vivo*, identify adhesion molecules and dendritic cells required for NK cell activation, and delineate the sequence of events for NK cell activation and protection against DENV infection.

## IMPORTANCE

Dengue is a mosquito-transmitted viral disease with a range of symptoms, from mild fever to life-threatening dengue hemorrhagic fever. The diverse disease manifestation is thought to result from a complex interplay between viral and host factors. Using mice engrafted with a human immune system, we show that human NK cells inhibit virus infection through secretion of the cytokine gamma interferon and reduce disease pathogenesis, including depletion of platelets and liver damage. During a natural infection, DENV initially infects dendritic cells in the skin. We find that NK cells interact with infected dendritic cells through physical contact mediated by adhesion molecules and become activated before they can control virus infection. These results show a critical role of human NK cells in controlling DENV infection *in vivo* and reveal the sequence of molecular and cellular events that activate NK cells to control dengue virus infection.

## INTRODUCTION

Dengue virus (DENV) infection is the most important arthropod-borne human viral disease worldwide. An estimated 390 million infections occur each year, of which 500,000 cases develop severe dengue and 20,000 die (1). DENV is a single-stranded RNA virus with four serotypes (DENV-1 to -4). Infection by any DENV serotype induces a spectrum of disease manifestations that range from nonspecific febrile illness to life-threatening dengue hemorrhagic fever/dengue shock syndrome (DHF/DSS). Multiple factors influence clinical outcomes, one of which appears to be a robust early host response to DENV infection that prevents the development of severe disease (2, 3).

Natural killer (NK) cells are a critical component of the innate immune system and provide early defense against viral infections (4, 5). Clinical and experimental studies have suggested a protective role of NK cells during the early stages of DENV infection (6–11). In adult patients, it was observed that mild dengue was correlated with higher numbers of circulating activated NK cells displaying cytotoxic and adhesion molecule profiles (6). Activated NK cells can inhibit viral infection through killing of virus-infected cells and secretion of gamma interferon (IFN-γ). Increased IFN-γ levels have been correlated with milder disease and higher survival rates in DHF patients (12, 13). In mouse models, DENV infection of immunocompetent C57BL/6J mice leads to peak IFN-γ production on day 5 postinfection, and NK cells represent the majority of cells that stain positive for intracellular IFN-γ (9, 10). The cytotoxicity of NK cells against DENV-infected cells was reported decades ago (14). NK cells also appear to play a key role in antibody-dependent cell-mediated cytotoxicity (ADCC) against DENV-infected cells during secondary infections when anti-DENV antibodies are present (15).

During a natural DENV infection by an infective mosquito, viral particles are introduced into the subcutaneous space, where resident dendritic cells (DCs) are probably the first cell type to be infected (16). A subsequent local inflammatory response likely recruits leukocytes, such as NK cells, to the site of infection. The migration of DENV-infected DCs into draining lymph nodes leads to systemic dissemination of DENV and infection of blood monocytes, which can become the dominant cell type infected by DENV (17). Recently, using a coculture system of monocyte-derived dendritic cells (MDDCs) and autologous human NK cells, it was reported that a combination of cell-cell contact and the secretion of type I interferon and tumor necrosis factor alpha (TNF-α) by DENV-infected MDDCs (iMDDCs) was required for NK cell activation, secretion of IFN-γ, and cytotoxicity toward iMDDCs (18). Despite these findings, how NK cells become activated during DENV infection and what molecules are involved in NK cell activation have not been fully elucidated.

By engrafting human CD34^+^ hematopoietic stem/progenitor cells into immunodeficient NOD-scid IL2rg^−/−^ (NSG) mice, it is possible to reconstitute the human immune system in the recipient mice (19, 20). We have previously shown that these humanized mice (humice) support robust DENV infection and subsequent pathogenesis, such as transient leukopenia and thrombocytopenia exclusively of human platelets (19). We have also shown that a single injection of plasmids encoding human interleukin 15 (IL-15) and Flt3 ligand (Flt3L) into humice dramatically increases the levels of human NK cell reconstitution and function (20). In this study, we have generated NK cell-optimized humice and shown that increased human NK cell reconstitution is associated with elevated levels of human IFN-γ at early stages of infection and reduced viremia and disease pathologies. Depletion of human NK cells or neutralization of IFN-γ abolishes the protection against DENV infection. Furthermore, using DENV-infected human monocytes and MDDCs, we show that NK cells are activated by DENV-infected MDDCs but not monocytes. NK cell activation is contact dependent and requires the engagement of adhesion molecules, such as LFA-1, DNAM-1, CD2, and 2β4. Taken collectively, the results of our study identify cell types and molecules and delineate the sequential events in NK cell activation and protection against DENV infection.

## RESULTS

### NK cells control acute dengue infection in humanized mice.

**FIG 1:**
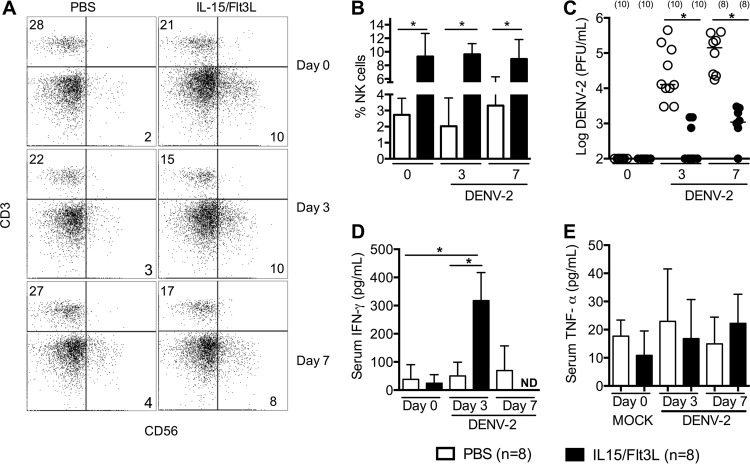
NK cells play a critical role in the control of dengue virus infection in humice. Humice were injected hydrodynamically with plasmids encoding the human cytokines IL-15 (50 μg) and Flt3L (50 μg). Seven days after cytokine treatment, mice were infected with 1 × 10^7^ PFU of DENV-2 by the intravenous route. (A) Representative human CD3 versus CD56 staining profiles, obtained by gating on human CD45^+^ cells before infection (day 0, i.e., 7 days after hydrodynamic plasmid injection) and at 3 and 7 dpi. Numbers indicate percentages of cells in gated regions. (B) Average percentages of CD3^−^ CD56^+^ NK cells among human CD45^+^ cells in blood of DENV-infected humice injected with IL-15 and Flt3L plasmids or PBS, determined at 0, 3, and 7 dpi. (C) Quantification of humouse viremia by plaque assay at 3 or 7 dpi. Each dot represents one humouse, numbers in parentheses indicate number of humice per group, and horizontal bars show median values. (D and E) Serum levels of human IFN-γ (D) and TNF-α (E) quantified by ELISA. Error bars in bar graphs show standard errors of the means (SEM). *, *P* < 0.05; ND, not detectable.

To study human NK cells in the control of acute DENV infection, we boosted the human NK cell levels in humanized mice by expressing human IL-15 and Flt3L through hydrodynamic injection of cytokine-encoding plasmids (20). Seven days after plasmid injection, the levels of human NK cells in the peripheral blood were increased from 2.7% ± 4.4% (*n* = 8) in uninjected humice to 8.6% ± 3.8% (*n* = 8) in injected humice (Fig. 1A and B). The elevated NK cell levels were maintained in plasmid-injected humice at 3 and 7 days following DENV-2 infection. In contrast, NK T cell levels were not significantly altered by either the expression of IL-15 and Flt3L or dengue infection in either uninjected humice (0.2% ± 0.1%, *n* = 8) or injected humice (0.3% ± 0.2%, *n* = 8). Humice injected with cytokine plasmids are referred to as NK-optimized humice hereinafter, whereas humice without cytokine plasmid injection are referred to control humice.

The effect of NK cells on DENV infection was determined by quantifying viral loads in the serum by plaque assay. As shown by the results in Fig. 1C, the levels of viremia increased from day 3 to day 7 following DENV-2 infection in both control and NK-optimized humice. However, viremia was approximately 100-fold lower in NK-optimized humice than in control humice both on day 3 and day 7 (*P* < 0.0001). The serum levels of human IFN-γ on day 3 postinfection were significantly higher in NK-optimized humice (310 ± 92 pg/ml, *n* = 10) than in control humice (50 ± 52 pg/ml) or mock-infected NK-optimized humice (38 ± 51 pg/ml) (Fig. 1D). Of note, the serum levels of human TNF-α were similar in infected NK-optimized humice (51 ± 58 pg/ml), infected control humice (55 ± 59 pg/ml), and mock-infected NK-optimized humice (48 ± 57 pg/ml) (Fig. 1E).

**FIG 2:**
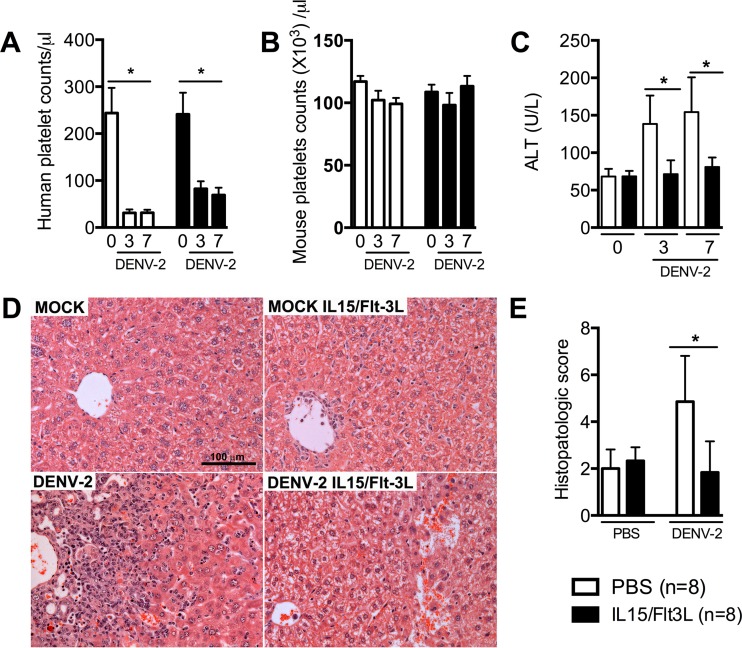
Dengue disease is less severe in NK-optimized humice. NK-optimized humice (7 days after hydrodynamic injection of plasmids) were infected with 1 × 10^7^ PFU of DENV-2 by the intravenous route. (A, B) Human and mouse platelet counts per microliter of blood of humice before infection (0) and at 3 and 7 dpi. (C) Serum levels of ALT in humice before infection and at 3 and 7 dpi. (D) Representative hematoxylin and eosin (H&E) staining of liver sections of mock- and DENV-2-infected mice at 7 dpi. Scale bar = 100 μm. (E) Semiquantitative analysis of hepatic damage based on H&E staining of liver sections of mock- and DENV-2-infected mice at 7 dpi. *, *P* < 0.05.

As commonly observed in dengue patients, DENV infection of humice leads to a drop in human platelets in both control and NK-optimized humice (Fig. 2A); however, the drop in human platelets in NK-optimized humice was less prominent than that in control humice (95 ± 8 platelets/µl blood versus 25 ± 3 platelets/µl blood; *P* < 0.05). Consistent with a previous report (19), DENV infection in humice did not significantly affect the levels of mouse platelets (Fig. 2B) or the overall hematocrit levels (data not shown). Hepatic damage, as assessed by serum alanine aminotransferase (ALT) levels, was significantly lower in NK-optimized humice than in control humice at both day 3 and day 7 postinfection (Fig. 2C). Histopathological analysis confirmed hepatic lesions induced by DENV-2 infection, as indicated by the presence of inflammatory infiltrates concentrated in the perivascular area spreading out into the liver parenchyma (Fig. 2D). The infiltrates were composed predominantly of mononuclear cells, including macrophages and multinucleated giant cells. Hepatic lesions with fibrous tissues and areas of necrosis and hemorrhage in the parenchyma were observed. Hepatocyte edema and degeneration were also evident in mice with severe lesions. The histopathological scores in the DENV-2-infected humice were 4.9 ± 1.8 out of a total of 20 points (Fig. 2E), whereas the scores were only 1.8 ± 1.2 points in infected NK-optimized humice. These results show that an elevated level of human NK cells is associated with reduced viremia and disease pathology in humice.

### NK cells control dengue infection through IFN-γ production in humice.

**FIG 3:**
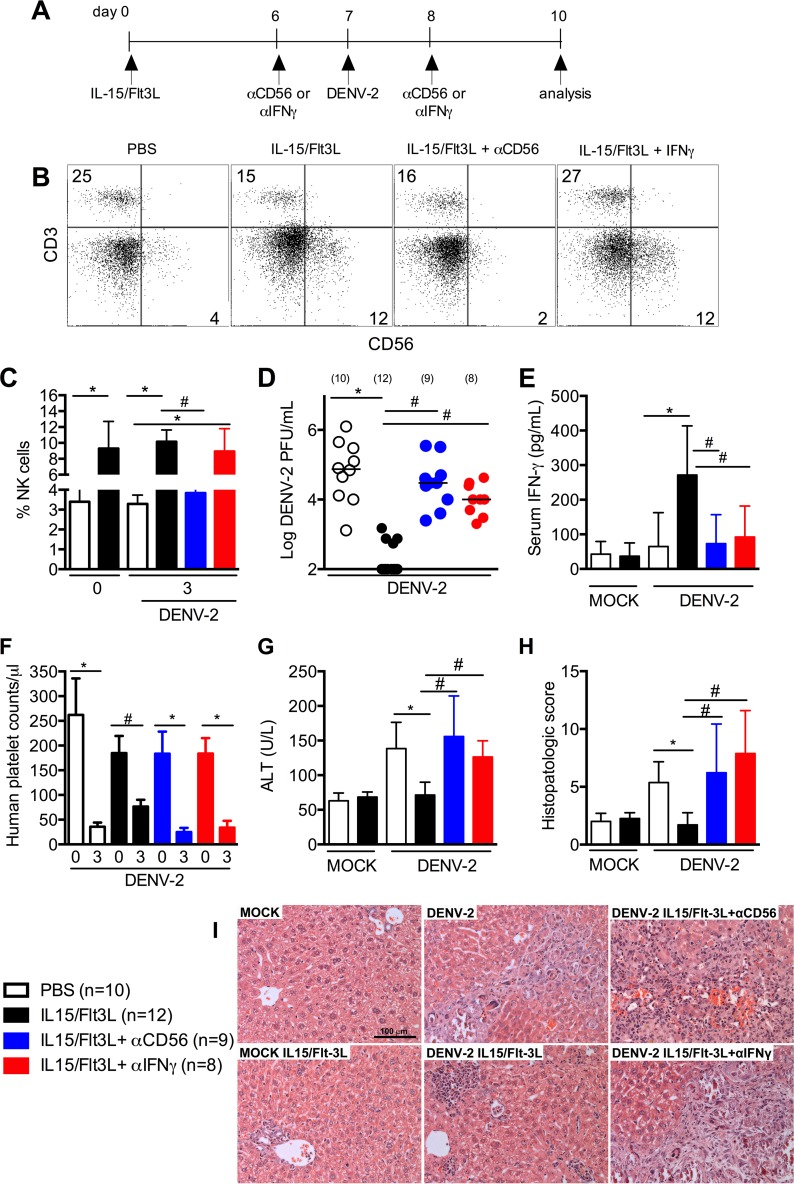
IFN-γ production by human NK cells is essential to control DENV infection and disease manifestation in humice. (A) Scheme of the experimental procedure. NK-optimized humice were injected with isotype, anti-human CD56, or anti-human IFN-γ antibody 1 day before and 1 day after infection with 1 × 10^7^ PFU DENV-2. Mice were sacrificed at 3 dpi. (B) Representative human CD3 versus CD56 staining profiles, obtained by gating on human CD45^+^ cells at 3 dpi. The numbers indicate percentages of cells in the gated regions. (C) Average percentages of CD3^−^ CD56^+^ NK cells within human CD45^+^ cell populations in the blood of humice. (D) Quantification of viremia at 3 dpi, determined by plaque assay. Each dot represents one humouse, and numbers in parentheses indicate number of humice per group. (E) Serum levels of human IFN-γ quantified by ELISA. (F) Human platelet counts per microliter of blood of humice. (G) ALT levels in sera of humice. (H) Semiquantitative analysis of hepatic damage at 3 dpi. (I) Representative H&E stains of liver sections from each group at 3 dpi. Scale bar = 100 µm. Error bars in bar graphs show standard errors of the means (SEM); horizontal bars in dot plot show median values. *, *P* < 0.05.

We determined the requirement of NK cells and IFN-γ in the control of DENV infection by depleting NK cells or neutralizing IFN-γ in NK-optimized humice. Humice were hydrodynamically injected with IL-15 and Flt3L plasmids (Fig. 3A). Six days later, the humice were given anti-CD56 antibody, anti-IFN-γ antibody, or control antibody. They were infected with DENV-2 on day 7 and given anti-CD56 antibody, anti-IFN-γ antibody, or control antibody again on day 8. Three days after infection (day 10 after hydrodynamic injection), humice were analyzed. With anti-CD56 antibody treatment, the levels of NK cells in NK-optimized humice were reduced from 9.8% ± 2.7% to 3.8% ± 1.2% (Fig. 3B and C), which was similar to the NK cell levels in control humice (3.7% ± 0.3%). Anti-IFN-γ antibody treatment did not significantly reduce the levels of NK cells in NK-optimized humice (9.54% ± 2.5%). Compared to the levels of viremia in NK-optimized humice, the levels in anti-CD56 antibody-treated NK-optimized mice were increased to the same levels as in control humice (Fig. 3D). Similarly, neutralization of IFN-γ also increased the viremia levels. Correspondingly, the serum IFN-γ levels were significantly reduced following both anti-CD56 and anti-IFN-γ antibody treatment in NK-optimized humice (Fig. 3E). Greater decreases in human platelets (Fig. 3F), higher levels of serum ALT (Fig. 3G), and more extensive liver damage (Fig. 3H and I) were observed in anti-CD56 and anti-IFN-γ antibody-treated NK-optimized humice. In contrast, neutralizing human TNF-α did not significantly increase the viremia (data not shown). These results show that NK cells play a critical role in controlling acute DENV infection in humice, at least partly through the secretion of IFN-γ.

### NK cells are activated by dengue virus-infected MDDCs.

To elucidate the mechanism by which NK cells control DENV infection, we explored *in vitro* coculture systems. We used dendritic cells as they are the first innate immune cell type that is naturally infected by DENV following a mosquito bite (21). Both NK cells and monocytes were isolated from the same peripheral blood mononuclear cell (PBMC) sample. Monocytes were cultured in the presence of IL-4 and granulocyte-macrophage colony-stimulating factor (GM-CSF) for 7 days to generate MDDCs. MDDCs were infected with DENV-2, and culture supernatants were harvested at 12, 24, 48, and 72 h to quantify virus titers and cytokine secretion. In four of five donors, the virus titer reached the peak level 48 h following infection (see Fig. S1A in the supplemental material). The levels of IFN-β, IL-12p40, and TNF-α in the supernatants were highest 48 h after infection (Fig. S2A to C), while the levels of IFN-γ peaked at 24 h after infection and significant levels remained at 48 h (Fig. S2D). To confirm the essential role of IFN-γ in controlling DENV infections, we added recombinant IFN-γ to cultures of infected MDDCs. Indeed, exogenous IFN-γ completely inhibited DENV replication in infected MDDCs (Fig. S3).

10.1128/mBio.00741-17.1 FIG S1  Kinetics of DENV-2 infection in monocyte-derived dendritic cells from heathy donors. Human monocytes were purified by negative selection, and monocyte-derived dendritic cells (MDDCs) were generated *in vitro* by culturing cells with IL-4 and GM-CSF for 7 days. MDDCs were then infected with DENV-2 (MOI of 1) for 2 h. After adsorption, cells were cultured and viral titers were measured by plaque assay in culture supernatants at 12, 24, 48, and 72 h. (A to E) Viral titers in culture supernatants from 5 different donors. Each dot represents a technical replicate, with median values indicated by horizontal lines. *, *P* < 0.05; ns, not statistically significant. Download FIG S1, TIF file, 0.4 MB. Copyright © 2017 Costa et al. 2017 Costa et al. This content is distributed under the terms of the Creative Commons Attribution 4.0 International license.

10.1128/mBio.00741-17.2 FIG S2  Cytokine levels in culture supernatants of monocyte-derived dendritic cells following DENV-2 infection. MDDCs were infected with DENV-2 (MOI = 1) for 2 h, and cytokine levels were measured by ELISA in culture supernatants at 0, 12, 24, 48, and 72 h. Mean values ± SD for 4 different donors are shown. *, *P* < 0.05; ND, not detectable. Download FIG S2, TIF file, 0.1 MB. Copyright © 2017 Costa et al. 2017 Costa et al. This content is distributed under the terms of the Creative Commons Attribution 4.0 International license.

10.1128/mBio.00741-17.3 FIG S3  Effects of different doses of recombinant IFN-γ on DENV-2 infection of monocyte-derived dendritic cells. MDDCs were infected with DENV-2 (MOI of 1) for 2 h. After adsorption, cells were incubated in the presence of medium or different doses of recombinant IFN-γ. Viral titers in culture supernatants were assayed by plaque assay 48 h after infection. Each dot represents a different donor, with median values indicated by horizontal lines. *, *P* < 0.05. Download FIG S3, TIF file, 0.02 MB. Copyright © 2017 Costa et al. 2017 Costa et al. This content is distributed under the terms of the Creative Commons Attribution 4.0 International license.

**FIG 4:**
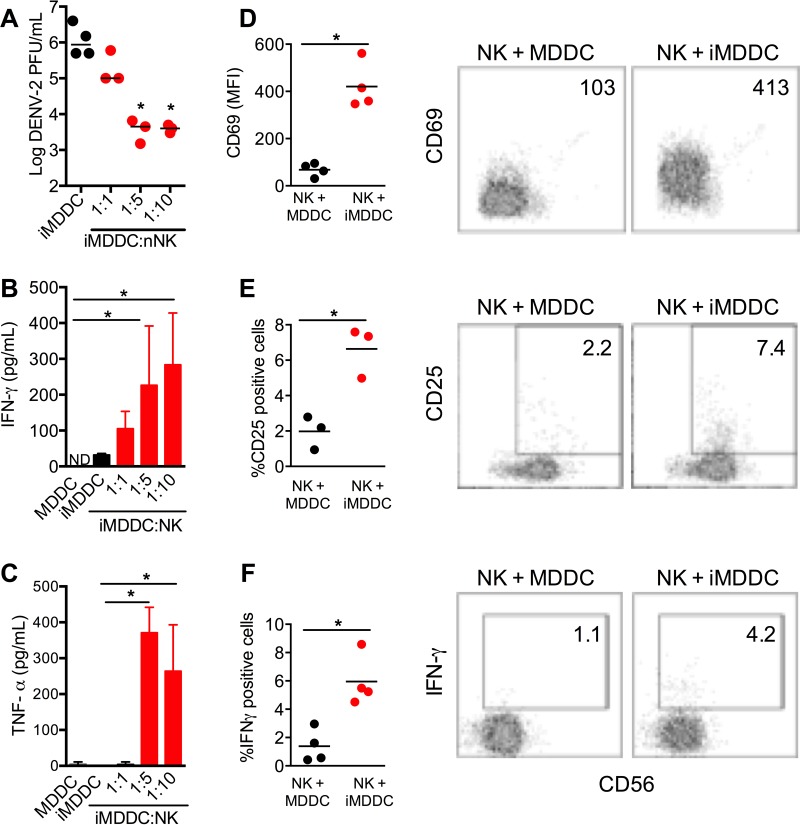
Naive NK cells are activated by infected MDDCs *in vitro*. MDDCs were infected with DENV-2 (MOI of 1) for 2 h and then incubated with autologous purified human NK cells for 48 h. (A to C) To determine the optimal MDDC/NK cell coculture ratio, infected MDDCs (iMDDCs) were cocultured with various numbers of NK cells. (A) Viral titers in culture supernatants as quantified by plaque assay. (B, C) IFN-γ (B) and TNF-α (C) levels in culture supernatants, quantified by ELISA. (D to F) Naive NK cells are activated after incubation with iMDDCs at a ratio of 1:5. (D) Representative CD56 versus CD69 staining profiles (right) and mean fluorescence intensities (MFI) of CD69 (left) for NK cells cocultured with uninfected MDDCs (NK + MDDC) or iMDDCs (NK + iMDDC). (E) Representative CD56 versus CD25 staining profiles (right) and percentages of CD25-positive NK cells (left) in cocultures with uninfected MDDCs or iMDDCs. (F) Representative CD56 versus IFN-γ staining profiles (right) and percentages of IFN-γ-positive NK cells (left) in cocultures with uninfected MDDCs or iMDDCs. Bar graphs show mean values ± standard deviations (SD). Numbers in flow cytometry plots indicate CD69 MFI or percentages of CD25- or IFN-γ-positive cells in gated regions. Each dot in a dot plot represents a different donor, and horizontal bars show median values. *, *P* < 0.05.

We cocultured NK cells and autologous MDDCs at ratios of 1:1, 1:5, and 1:10. At a ratio of 1:5, NK cells inhibited DENV-2 production by ~100-fold (Fig. 4A). The level of IFN-γ in the culture supernatants was significantly increased compared to the level in supernatants from infected MDDCs (iMDDCs) only (Fig. 4B). Likewise, the level of TNF-α was also significantly increased at a ratio of 1:5 (Fig. 4C). Activation markers like CD69 and CD25 were upregulated on NK cells after coculture for 48 h with infected MDDCs compared to their levels on NK cells cocultured with uninfected MDDCs (Fig. 4D and E). Consistent with the IFN-γ levels detected by enzyme-linked immunosorbent assay (ELISA), the percentages of NK cells positive for intracellular IFN-γ were significantly increased following coculture with infected MDDCs compared with the percentages in uninfected MDDCs (6.0% ± 0.9% versus 1.4% ± 0.6%, *P* < 0.01) (Fig. 4F). Furthermore, the ability of NK cells to control DENV infection was not restricted to the DENV-2 serotype. Coculture of naive NK cells with MDDCs infected with DENV-1, DENV-3, or DENV-4 also resulted in an ~100-fold reduction in viral titer in the culture supernatants (Fig. S4A) and elevated IFN-γ production (Fig. S4B). These results suggested that NK cells are activated after coculture with DENV-infected MDDCs, leading to secretion of IFN-γ and TNF-α, which control replication in infected MDDCs.

10.1128/mBio.00741-17.4 FIG S4  Naive NK cells control DENV-1, DENV-3, and DENV-4 infection of MDDCs *in vitro*. MDDCs were infected with DENV-1, DENV-3, or DENV-4 (MOI or 1) for 2 h and then incubated with autologous purified human NK cells at a ratio of 1:5 for 48 h. (A) Viral titers in the culture supernatants, measured by plaque assay. Each dot represents a different donor. (B) IFN-γ levels (mean values ± SD) in culture supernatants, determined by ELISA. *, *P* < 0.05; ND, not detectable. Download FIG S4, TIF file, 0.1 MB. Copyright © 2017 Costa et al. 2017 Costa et al. This content is distributed under the terms of the Creative Commons Attribution 4.0 International license.

### Cell-cell contact is required for NK cell activation and control of DENV-2 infection in MDDCs.

**FIG 5:**
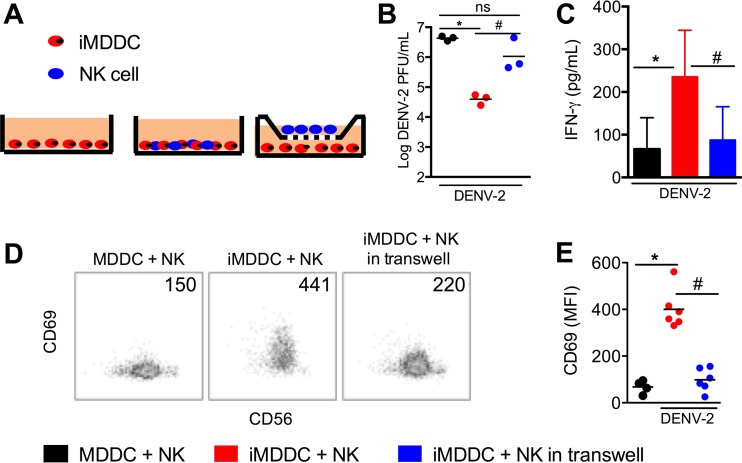
Cell-cell contact is required for efficient control of DENV infection of MDDCs *in vitro*. (A) Scheme of the transwell experiment setup. (B) Viral titers in the culture supernatants at 48 h of infection. (C) IFN-γ levels in the culture supernatants at 48 h of infection. (D) Representative CD56 versus CD69 staining profiles of NK cells at 48 h of infection. Numbers indicate MFI. (E) MFI of CD69 on NK cells in three cocultures at 48 h of infection. Bar graph shows mean values ± SD. Each dot in a dot plot represents a different donor, and horizontal bars show median values. *, *P* < 0.05.

We determined whether cell-cell contact was required for NK cell activation by infected MDDCs via a transwell system. NK cells were either cocultured with infected autologous MDDCs in the same chamber or separated by a 0.4-μm membrane in a different chamber where NK cells did not have direct contact with infected MDDCs (Fig. 5A). As expected, the virus titers in the supernatants were reduced by ~100-fold when NK cells were cocultured with infected MDDCs compared to the titers in infected MDDCs alone (Fig. 5B). The reduction of virus titers was mostly abolished when NK cells were placed in the transwell. Consistently, NK cells were activated to express much higher levels of CD69 and IFN-γ (Fig. 5C to E) when they were cocultured with infected MDDCs in the same chamber. Although CD69 was also upregulated on NK cells when they were cocultured with infected MDDCs in transwells, the level of upregulation was much lower. Thus, direct cell-cell contact between NK cells and infected MDDCs is a critical first step in which NK cells become activated to secrete IFN-γ and control DENV infection.

### Adhesion molecules are required for interactions between NK cells and infected MDDCs.

Given the requirement for direct cell-cell contact in NK cell activation and control of dengue infection, we examined the involvement of specific NK cell surface molecules, including adhesion molecules (2β4, LFA-1, DNAM-1, and CD2) and natural cytotoxicity-triggering receptors (NKp30, NKp44, NKp46, and NKG2D). NK cells expressed the various adhesion molecules, but their levels of expression were not changed significantly following coculture with infected MDDCs (Fig. S5). NK cells expressed NKp46 and NKG2D but very little, if any, NKp44 and NKp30. The expression of these receptors was not altered following coculture of NK cells with infected MDDCs (Fig. S6).

10.1128/mBio.00741-17.5 FIG S5  Expression of adhesion molecules by NK cells before and after coculture with infected MDDCs. Human NK cells were cocultured with DENV-2-infected autologous MDDCs for 48 h. Cells were stained with antibodies specific for CD56, CD1a, and the indicated adhesion molecules. Shown are representative staining profiles of CD56 versus the indicated adhesion molecules, obtained by gating on CD1a-negative cells (left). Numbers are percentages of positive cells in gated regions. Percentages of positive cells (mean values ± SEM) from four donors are shown on the left. Each dot represents a different donor. Download FIG S5, TIF file, 0.2 MB. Copyright © 2017 Costa et al. 2017 Costa et al. This content is distributed under the terms of the Creative Commons Attribution 4.0 International license.

10.1128/mBio.00741-17.6 FIG S6  Surface expression of NK cell receptors before and after coculture with infected MDDCs. Human NK cells were cocultured with DENV-2-infected autologous MDDCs for 48 h. Cells were stained with antibodies specific for CD56, CD1a, and the indicated NK cell receptors. Shown are representative staining profiles of CD56 versus the indicated receptors, obtained by gating on CD1a-negative cells (left). Numbers are percentages of positive cells in gated regions. Percentages of positive cells (mean values ± SEM) from five donors are shown on the right. Each dot represents a different donor. Download FIG S6, TIF file, 0.2 MB. Copyright © 2017 Costa et al. 2017 Costa et al. This content is distributed under the terms of the Creative Commons Attribution 4.0 International license.

**FIG 6:**
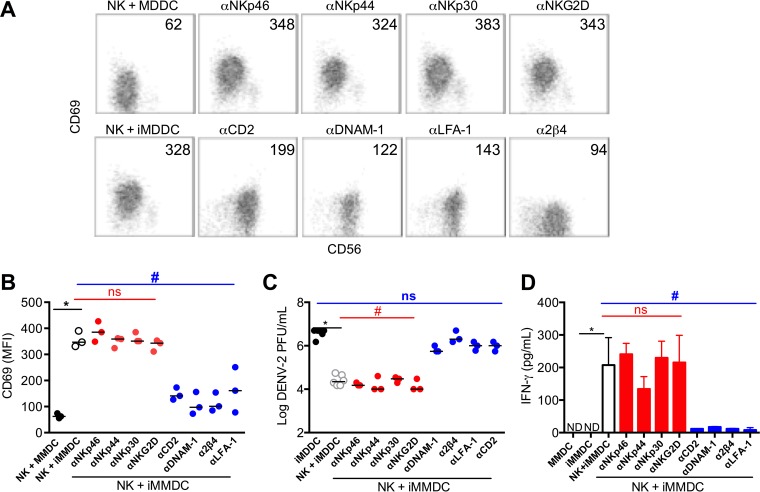
Adhesion molecules on NK cell surface are involved in NK cell-MDDC interactions. Human monocytes were purified by negative selection, and monocyte-derived dendritic cells (MDDCs) were generated *in vitro* by adding IL-4 and GM-CSF for 7 days. Autologous NK cells were purified by negative selection. MDDCs were infected with DENV-2 (MOI of 1) for 2 h and then incubated with purified autologous NK cells for 48 h in the presence or absence of anti-NK cell receptor antibodies. (A) Shown are representative CD69 versus CD56 staining profiles in the presence or absence of blocking antibody. (B) Mean fluorescence intensities (MFI) of CD69 on NK cells in coculture with iMDDCs in the presence or absence of blocking antibody. (C) Viral loads in culture supernatants after 48 h, determined by plaque assay. (D) IFN-γ levels in culture supernatants after 48 h, determined by ELISA. Each dot in a dot plot represents a different donor, and horizontal lines show median values. Bar graph shows mean values ± SD. ND, not detectable; ns, not statistically significant; *, *P* < 0.05.

To block each of these receptors, purified NK cells were incubated with receptor-specific antibodies individually for 2 h and then cocultured with infected MDDCs for 48 h. NK cells were assayed for CD69 expression, and the culture supernatants for virus titers and IFN-γ. As shown by the results in Fig. 6, blocking NKp46, NKp44, NKp30, and NKG2D did not inhibit NK cell activation or reduce the virus titer; i.e., NK cells were activated in the presence of these blocking antibodies as indicated by upregulation of CD69 (Fig. 6A and B), reduction of virus titers by about 100-fold (Fig. 6C), and high levels of IFN-γ in the culture supernatants (Fig. 6D). In contrast, blocking the adhesion molecules CD2, DNAM-1, LFA-1, and 2β4 all resulted in inhibition of NK cell activation (Fig. 6A and B), elevated virus titers (Fig. 6C), and diminished IFN-γ secretion (Fig. 6D). Blocking all four adhesion molecules simultaneously did not result in an additive effect on the reduction of viral titers (data not shown). These results show that adhesion molecules but not specific natural cytotoxicity receptors are required for NK cell interaction with infected MDDCs, consistent with a requirement for cell-cell contact for NK cell activation, IFN-γ secretion, and control of DENV infection.

### Activated NK cells also control DENV infection in human monocytes.

**FIG 7:**
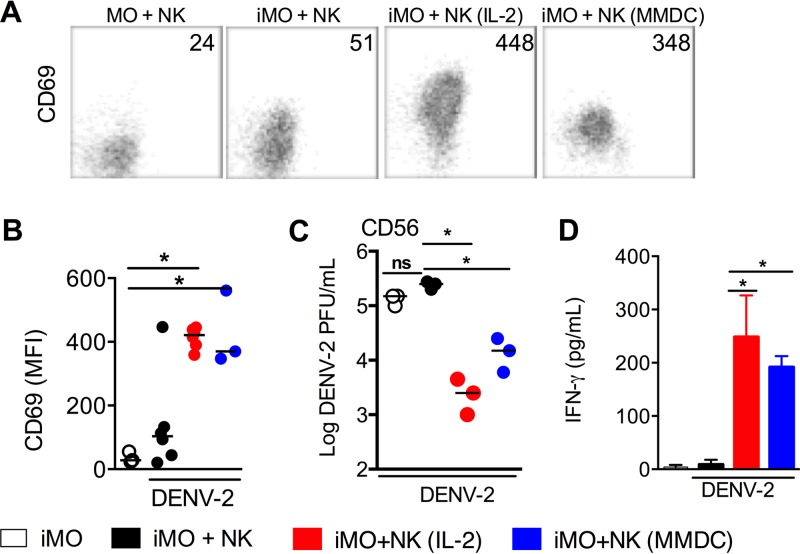
Activated NK cells also control DENV infection in human monocytes. Human NK cells and monocytes (MO) were purified from PBMC by negative selection. NK cells were activated by culturing with either IL-2 for 5 days or iMDDCs for 48 h. Activated NK cells from iMDDC culture were purified by negative selection. Purified monocytes were infected with DENV-2 at an MOI of 10 for 2 h (iMO) and then cocultured with naive, IL-2-activated, or iMDDC-activated autologous NK cells for 48 h. NK cells were assayed for CD69 expression, and culture supernatants were assayed for viral titers and IFN-γ. (A) Representative CD56 versus CD69 staining profiles, obtained by gating on CD1a-negative cells from the four different cocultures. The numbers indicate CD69 MFI. (B) CD69 MFI of NK cells in the four cocultures. (C) Viral titers in the four culture supernatants. (D) IFN-γ levels in the four culture supernatants. Each dot in the dot plots represents a different donor, and horizontal bars show median values. Bar graph shows mean values ± SD. *, *P* < 0.05; ND, not detectable; ns, not statistically significant.

Monocytes and macrophages can also be infected by DENV (17). We also tested whether infected monocytes can activate NK cells. Both NK cells and monocytes were purified from human PBMC. NK cells were cocultured with autologous DENV-infected monocytes at a multiplicity of infection (MOI) of 10 for 48 h, and NK cell activation, IFN-γ secretion, and virus titers were assayed. As shown by the results in Fig. 7, NK cells did not upregulate CD69, reduce viral titers in the culture supernatants, or secrete IFN-γ. In contrast, if NK cells were activated with IL-2 (22, 23) prior to their addition into the coculture with infected monocytes, the virus titers were reduced by about 100-fold concomitant with a significant level of IFN-γ being found in the culture supernatants.

During a natural DENV infection in humans, dendritic cells in the skin are infected first, followed by infection of monocytes and macrophages in the peripheral blood. We investigated whether NK cells activated by infected MDDCs could control DENV infection in human monocytes. NK cells were cocultured with infected MDDCs for 48 h, purified by cell sorting, and cultured with DENV-infected autologous monocytes at a ratio of 1:5 for another 48 h. As with IL-2-activated NK cells, iMDDC-activated NK cells upregulated CD69 expression (Fig. 7A and B), reduced the virus titers by 10-fold, compared to 100-fold by IL-2-activated NK cells (Fig. 7C), and secreted IFN-γ (Fig. 7D). Similar to what we observed in NK cell-MDDC coculture, cell-cell contact (Fig. S7) and adhesion molecules (Fig. S8) were required for the reduction of viral titers and IFN-γ secretion by NK cells cocultured with DENV-infected monocytes. Thus, NK cells are not activated directly by DENV-infected monocytes, but if NK cells are first activated by DENV-infected MDDCs, they can control DENV infection in monocytes.

10.1128/mBio.00741-17.7 FIG S7  Cell-cell contact is required for control of DENV-2 infection in monocytes *in vitro*. Human monocytes and NK cells were purified by negative selection. NK cells were activated by culturing with IL-2 for 5 days. Monocytes were infected with DENV-2 (MOI of 10) for 2 h and incubated with either naive NK cells or IL-2-activated NK cells for 48 h in the same chamber or in a transwell. (A) Viral titers in culture supernatants. (B) IFN-γ levels in culture supernatants. Bar graph shows mean values ± SD. Each dot in a dot plot represents a different donor. *, *P* < 0.05; ND, not detectable. Download FIG S7, TIF file, 0.1 MB. Copyright © 2017 Costa et al. 2017 Costa et al. This content is distributed under the terms of the Creative Commons Attribution 4.0 International license.

10.1128/mBio.00741-17.8 FIG S8  Adhesion molecules are involved in NK cell-monocyte interactions and control of infection. Human monocytes and NK cells were purified from PBMC by negative selection. NK cells were activated by culturing with IL-2 for 5 days. Monocytes were infected with DENV-2 (MOI of 10) for 2 h and incubated with IL-2-activated NK cells for 48 h in the presence or absence of various neutralizing antibodies. (A) Viral titers in culture supernatants. (B) IFN-γ levels in culture supernatants. Bar graphs show mean values ± SD. Each dot in a dot plot represents a different donor, and horizontal bars show median values. *, *P* < 0.05; ND, not detectable; ns, not statistically significant. Download FIG S8, TIF file, 0.1 MB. Copyright © 2017 Costa et al. 2017 Costa et al. This content is distributed under the terms of the Creative Commons Attribution 4.0 International license.

## DISCUSSION

In this study, we have examined the role of human NK cells in the control of DENV infection *in vivo* (NK-optimized humice) and the cellular and molecular events required for NK cell activation and IFN-γ secretion. Although studies have suggested an important role of NK cells and IFN-γ in protection against DENV infection, previous studies were carried out either *in vitro* using purified NK cells due to the lack of a small animal model of dengue infection (6, 7, 24) or through correlative studies in patients (6). We have taken advantage of NK-optimized humice that support robust DENV infection. By modulating the level of human NK cells or IFN-γ in humice and examining the effects on viremia and disease pathologies, our results confirmed an essential role played by human NK cells and IFN-γ in the control of DENV infection *in vivo*. Our study specifically shows that the NK cell response to DENV infection in humice is primarily mediated by secretion of IFN-γ.

More importantly, we have identified molecules required for NK cell activation and IFN-γ secretion. We showed that cell-cell contact between infected MDDCs and NK cells is critical for NK cell activation and IFN-γ secretion. By blocking specific NK cell receptors, we identified adhesion molecules, including CD2, 2β4, LFA-1, and DNAM-1, on NK cells, but not NK cell activation receptors, as required for mediating the cell-cell contact and NK cell activation. Interestingly, DENV infection did not change the expression of the ligands of these receptors, including CD58, CD48, ICAM-1, and CD155, on infected MDDCs (data not shown), consistent with a similar report by Lim et al. (18). In addition, blocking any of these four adhesion molecules almost completely abolished NK cell activation and IFN-γ expression, whereas blocking all four simultaneously did not show any additive effect, suggesting cooperative engagement of these adhesion molecules in NK activation and IFN-γ secretion. We have shown previously that the activation of NK cells by *Plasmodium falciparum*-infected red blood cells also requires adhesion molecules LFA-1 and DNAM-1, but not CD2 and 2B4, in a contact-dependent manner (25). These findings suggest a general mechanism of cell-cell contact involving adhesion molecules in pathogen-mediated NK cell activation, but the specific adhesion molecules involved may differ with different pathogens.

In contrast to the case for adhesion molecules, blocking NKp44, NKp46, NKp30, and NKG2D did not have any effect on NK cell activation and IFN-γ secretion and control of DENV infection. The levels of NKp44 and NKp30 on NK cells are minimal, and this could explain the absence of any effect. Both NKG2D and NKp46 are expressed by NK cells. In fact, genome-wide associated studies have identified MICA and MICB, which are ligands for NKG2D, as susceptibility loci for dengue shock syndrome (26, 27). However, blocking NKG2D has no effect on the NK cell response to DENV2-infected MDDCs or monocytes. A similar observation has also been reported with human cytomegalovirus (HCMV) infection, where inhibition of surface expression of ULBPs, a class of NKG2D ligands, did not affect NK cell activation, suggesting the presence of NKG2D-independent pathways (28).

Our results are consistent with previous findings showing a critical role of IFN-γ in NK cell-mediated antiviral responses. When NK cells were cocultured with DENV-infected MDDCs, secretion of both IFN-γ and TNF-α was induced. However, DENV infection of humanized mice induced IFN-γ but not TNF-α in the circulation. Furthermore, neutralizing IFN-γ but not TNF-α in humanized mice abolished the NK cell response to DENV, suggesting that IFN-γ but not TNF-α is required for NK cell control of DENV infection *in vivo*. Lim et al. have reported that the combination of cell-cell contact and type I interferon and TNF-α is required for activating NK cell cytotoxicity (18). However, a closer examination of their data revealed that cell-cell contact contributes nearly 90% to NK cell activation. Consistent with this, the results from our transwell study show that cell-cell contact plays a dominant role in NK cell activation, whereas the effect of soluble factors is minimal.

Our study also shed light on the possible sequence of events that leads to NK cell activation and control of DENV infection *in vivo*. During a natural dengue infection, resident DCs in the skin are the first cell type to be infected (16). Migration of infected DCs into the draining lymph nodes is thought to spread the infection systemically, leading to infection of monocytes in the circulation (17). It is notable that infected MDDCs, but not monocytes, activate NK cells and IFN-γ secretion, suggesting that NK cells are likely activated at a very early stage of DENV infection. Following the infection of DCs, the ensuing inflammatory response recruits leukocytes, such as NK cells, into the site of infection. NK cells could be activated at the site of the mosquito bite or in the draining lymph nodes. Activated NK cells and their secreted IFN-γ then control infection of DCs, as well as monocytes. It would be interesting to elucidate the molecular basis underlying the difference by which infected MDDCs but not monocytes activate NK cells.

## MATERIALS AND METHODS

### Ethics statement.

This study was carried out in strict accordance with the ethical and animal experiment regulations of the National Advisory Committee for Laboratory Animal Research (NACLAR) (29). The experimental protocol was approved by the Institutional Animal Care and Use Committee (IACUC) of the National University of Singapore (NUS) and Massachusetts Institute of Technology (MIT) under access number R13-6157. All surgeries were performed under 5% isoflurane anesthesia, and all efforts were made to minimize animal suffering. Studies with DENV were conducted under biosafety level 2 (BSL2) containment at the National University of Singapore animal facility. Human CD34^+^ hematopoietic stem/progenitor cells were purified from fetal liver by CD34^+^ magnetic selection (Stem Cell Technologies). All women were informed of the risks of participation in the study, and a written consent for fetal tissue donation to research was obtained from each woman. SingHealth and National Health Care Group Research Ethics Committees Singapore specifically approved this study (CIRB reference number 2012/064/B).

### Generation of humice and NK-optimized humice.

Nod-Scid-IL-2Rγ^null^ (NSG) mice were purchased from Jackson Laboratories (Bar Harbor, ME) and maintained under pathogen-free conditions in National University of Singapore animal facilities. To generate humice, 2 × 10^5^ human CD34^+^ cells were injected intracardially into each sublethally irradiated NSG pup within 24 to 48 h of birth as previously described (20). At 10 to 12 weeks, humice were assessed for human leukocyte reconstitution. Those with more than 40% human leukocyte reconstitution in the peripheral blood mononuclear cells (PBMC) were further injected hydrodynamically with plasmids encoding human IL-15 (50 μg) and Flt3L (50 μg) to generate NK-optimized humice. Anti-human CD56 (HCD56), anti-human IFN-γ, and anti-human TNF-α antibodies were injected intravenously at 50 µg/mouse to deplete human NK cells or neutralize human IFN-γ and human TNF-α, respectively, 24 h before and 24 h after dengue infection (25). To assess for NK cell depletion, anti-human CD56 antibody (MEM188), which recognizes a different epitope from anti-human CD56 antibody (HCD56), was used.

### Production of viruses and plaque assay.

DENV-2 (Eden-07K2861), DENV-1 (Eden-2402DK1), DENV-3 (Eden-863DK1), and DENV-4 (Eden-2270DK1) strains were propagated in mosquito cells (*Aedes albopictus* clone C6/36 [ATCC CRL-1660, existing collection in our laboratory]) in RPMI 1640 medium (Gibco) with 10% fetal bovine serum (FBS) (Lonza) at 28°C. To concentrate the virus, 50 ml of the cell culture supernatant was loaded onto a VivaCell 100 centrifugal concentrator (Sartorius) and centrifuged at 2,000 × *g* for 10 min, and the supernatant remaining in the concentrator was aliquoted and stored at −80°C. For quantification of virus, BHK-21 cells (C-13) (ATCC CCL-10; originally from ATCC, existing collection in our laboratory) were grown to a confluent monolayer in RPMI 1640 medium with 10% FBS (Lonza) and 1% penicillin-streptomycin-glutamine (Gibco) in 24-well plates at 37°C. The virus was serially diluted in serum-free medium and then inoculated into the cells at 37°C with gentle shaking every 15 min for 1 h. Next, the medium was replaced with RPMI 1640 medium containing 2% carboxymethylcellulose (CMC) and 2% FBS (Lonza) (overlay medium) and the culture kept at 37°C. After 5 days, the cells were fixed in 3% formalin in phosphate-buffered saline (PBS) for 1 h, washed, and stained with 0.1% crystal violet in 10% formalin solution for 1 h. Then, the plates were washed with water, and the plaques were counted. One PFU of the cell culture supernatant was observed to have close to 1,000 copies of viral RNA.

### Infection of humanized mice.

Humanized mice were infected by injecting 1 × 10^7^ PFU of the concentrated DENV-2 virus in 200 µl of RPMI 1640 medium through the tail vein. Control humanized mice reconstituted with the same batch of human CD34^+^ fetal liver cells were injected with 200 µl of plain RPMI 1640 medium. In some experiments, virus was heat inactivated (at 60°C for 1 h) and then injected into the humanized mice as the control. Blood, liver, spleen, lymph node (axillary), and bone marrow (femur) were collected from control and infected mice at different days postinfection (dpi) for flow cytometry assays, viral RNA quantification, alanine aminotransferase (ALT) assays, platelet counts, and histology.

### ALT measurement and histopathological analysis.

ALT levels in the sera from mice at 7 dpi were measured using a Cobas C111 analyzer (Roche) in the NUS comparative medicine in-house veterinary diagnostic laboratory. Histopathological analysis was performed as previously described by Costa et al. (10). Briefly, liver samples from humice were obtained at day 7 after DENV-2 inoculation. Afterward, samples were immediately fixed in 10% buffered formalin for 24 h and embedded in paraffin. Tissue sections (4 mm thick) were stained with hematoxylin and eosin (H&E) and evaluated under an Axioskop 40 microscope (Carl Zeiss, Göttingen, Germany) adapted to a digital camera (PowerShot A620; Canon, Tokyo, Japan). Histopathology scoring was carried out by a pathologist in a blinded fashion according to a set of custom-designed criteria described by Costa et al. (10). Hepatocyte swelling, degenerative changes, necrosis, and hemorrhage were each graded on a scale of 0 to 5 (0, absent; 1, minimal; 2, slight; 3, moderate; 4, marked; and 5, severe). The sum of the score was computed to derive the overall liver damage score. A total of two sections for each animal were examined.

### NK cell and MDDC/monocyte coculture system.

Monocytes were negatively selected from total PBMC using the EasySep human monocyte enrichment kit without CD16 depletion (Stemcell Technologies). To generate MDDCs, monocytes were cultured for 7 days in RPMI 1640 (Sigma) supplemented with 10% FBS (Gibco), IL-4 (500 U/ml; PeproTech), and GM-CSF (500 U/ml; PeproTech). The MDDC phenotype was verified by flow cytometry as CD1a^+^ CD209^+^ CD14^−^ CD83^−/low^. Infection of MDDCs by DENV-2 virus was performed at an MOI of 1 in RPMI medium (RPMI supplemented with 3% FBS) for 2 h, followed by 3 washes to remove free virus. Autologous NK cells were purified from PBMC using the EasySep human NK cell enrichment kit (Stemcell Technologies) and cocultured with infected or uninfected MDDCs at ratios of 1:1, 1:5, and 1:10 in RPMI medium.

Alternatively, monocytes were infected with DENV (MOI of 10) in RPMI medium (RPMI supplemented with 3% FBS) for 2 h, followed by 3 washes to remove free viruses. A higher MOI was used for monocyte infection than for MDDC infection because monocytes are more resistant to DENV infection. Autologous NK cells were cocultured with infected or uninfected monocytes at ratios of 1:1, 1:5, and 1:10 in RPMI medium. For transwell experiments, culture plates with a 0.4-μm membrane from Millipore were used. For receptor blocking experiments, the following neutralizing antibodies (BioLegend) were used at 1 μl/ml: anti-CD11a (HI111), anti-CD18 (TS1/18), anti-CD2 (TS1/8), anti-DNAM (11A8), anti-2B4 (C1.7), anti-NKp46 (9E2), anti-NKp30 (P30-15), anti-NKp44 (P44-8), and anti-NKG2D (1D11) antibodies.

### Measurement of cytokine/chemokine concentrations.

The concentrations of cytokines (TNF-α, IFN-β, IFN-γ, IL-12p40, and IL-18) in serum samples or culture supernatants were measured using commercially available antibodies and according to the procedures supplied by the manufacturer (R&D Systems, Minneapolis, MN). The results are expressed as picograms per milliliter. The detection limits of the ELISA assays were in the range of 4 to 8 pg/ml.

### Flow cytometry.

Antibodies specific for human CD45 (2D1), CD14 (HCD14), CD56 (HCD56), CD56 (MEM188), CD19 (HIB19), CD3 (HIT3a), CD69 (FN50), and CD25 (FN50) and for mouse CD45.1 (A20) were obtained from BioLegend. Cells were stained with appropriate antibodies in 50 μl of fluorescence-activated cell sorting (FACS) buffer (PBS with 0.2% bovine serum albumin [BSA] and 0.05% sodium azide) for 30 min on ice. Stained cells were analyzed by flow cytometry using an LSRII instrument, and data were analyzed by using FACSDiva (BD Biosciences) or FlowJo (TreeStar, Inc.).

Mouse and human platelets were evaluated as previously described by Sridharan et al. (19). Briefly, 10 µl of whole blood was resuspended in 600 µl of FACS buffer. Antibodies specific for human CD41 and mouse CD41 were added and incubated for 10 min. Then, 25 µl of CountBright absolute counting beads (Invitrogen) was added to each sample and analyzed until 1,000 beads were recorded per sample. The number of human or mouse platelets was calculated as follows: count of platelets/µl of whole blood = (target platelet events/bead events) × (bead count/10 µl).

### Statistical analysis.

For numerical continuous data, such as mean fluorescence intensities (MFI), cell percentages, DENV viremia, cytokine concentrations, liver enzymes, and platelet counts, the unpaired *t* test was used to determine whether the means of the two samples were significantly different. For ranked data, such as the histological scoring of liver damage, the Mann-Whitney test was used to determine whether the scores were significantly different. Statistical analysis was performed using GraphPad Prism (GraphPad Software, Inc.).
