# Supplementary figures and images for: Dengue Virus-Infected Dendritic Cells, but Not Monocytes, Activate Natural Killer Cells through a Contact-Dependent Mechanism Involving Adhesion Molecules
Source: mBio. 2017 Aug 1;8(4):e00741-17. doi: 10.1128/mBio.00741-17 (PMC5539423; doi:10.1128/mBio.00741-17)

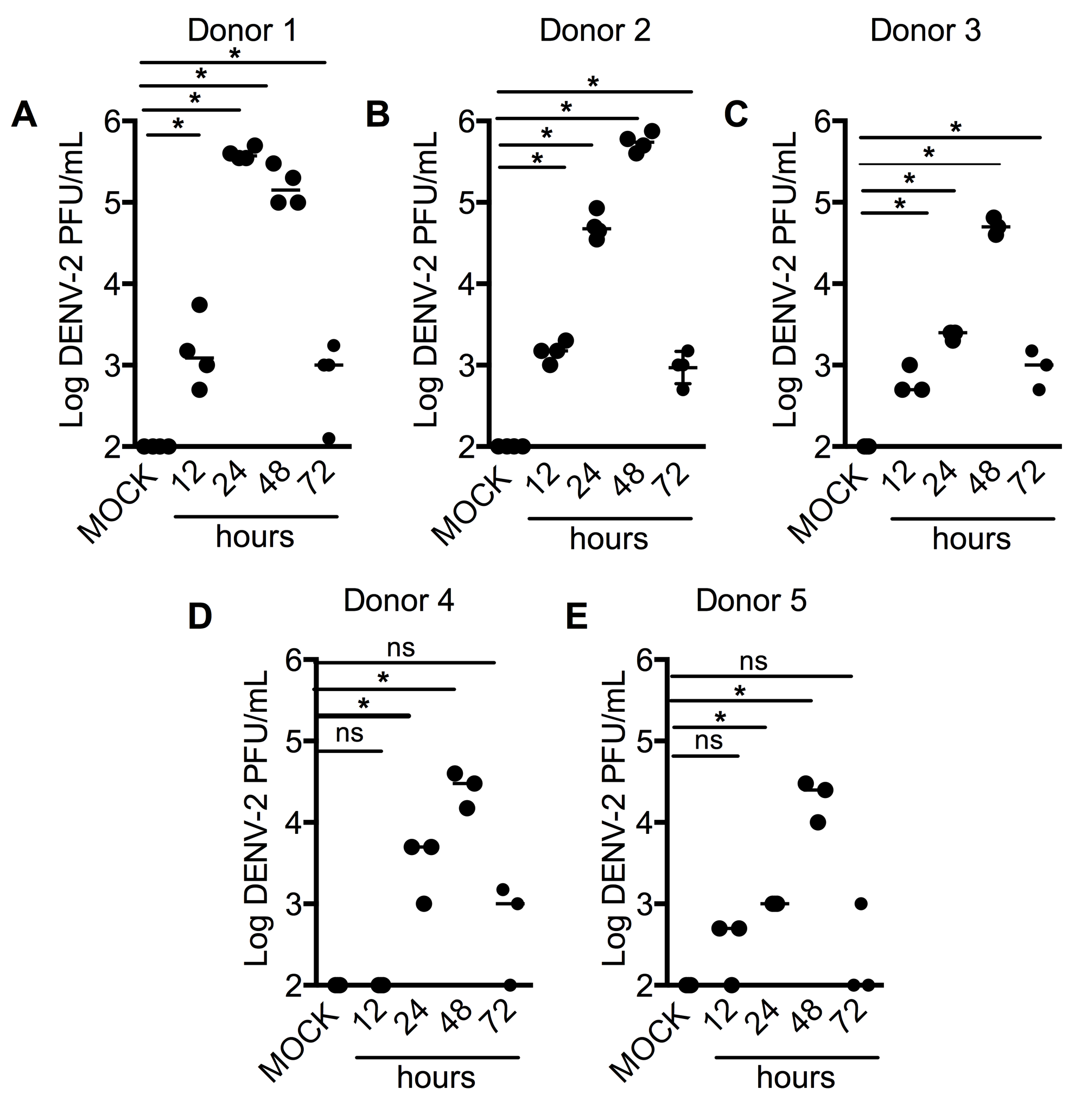

Supplement: FIG S1 [file mbo004173407sf1.tif]

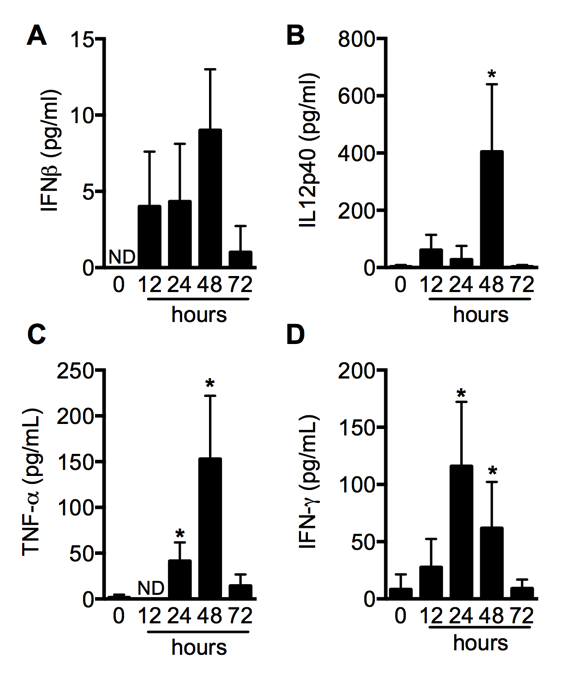

Supplement: FIG S2 [file mbo004173407sf2.tif]

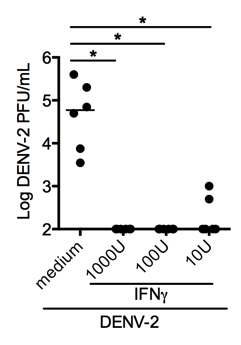

Supplement: FIG S3 [file mbo004173407sf3.tif]

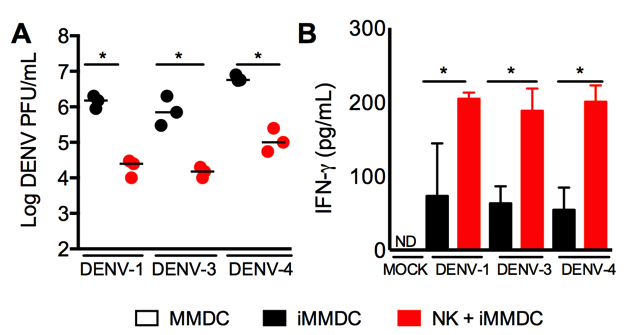

Supplement: FIG S4 [file mbo004173407sf4.tif]

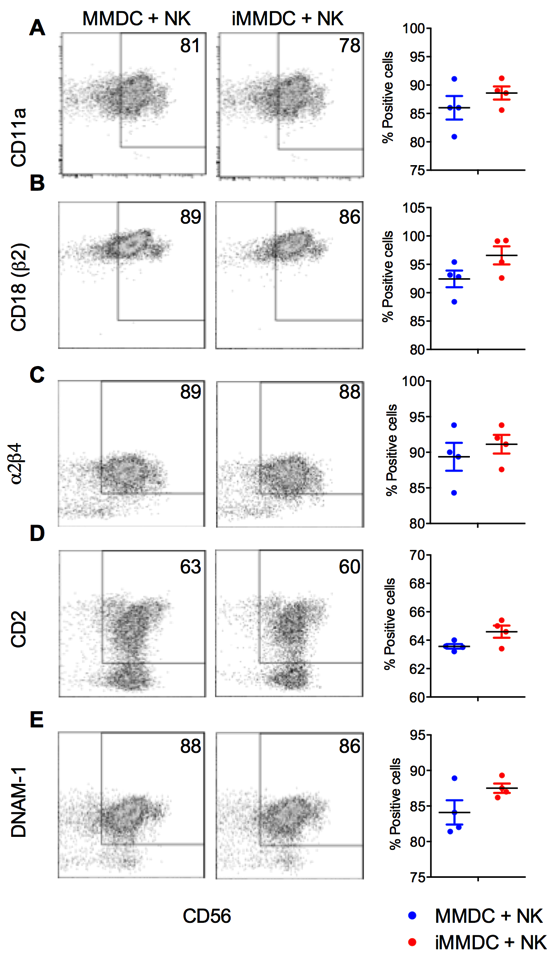

Supplement: FIG S5 [file mbo004173407sf5.tif]

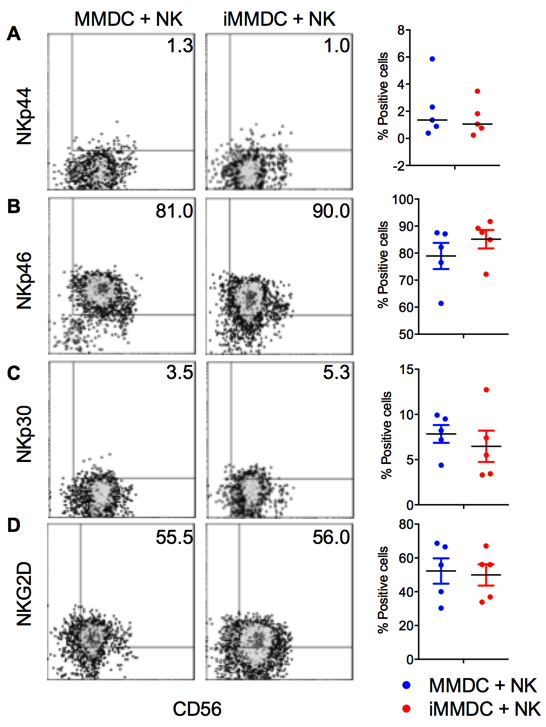

Supplement: FIG S6 [file mbo004173407sf6.tif]

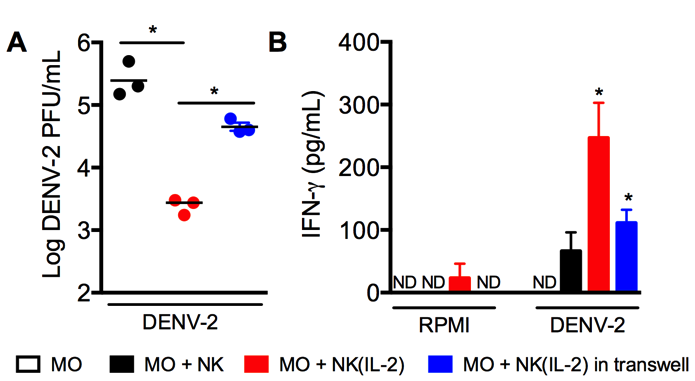

Supplement: FIG S7 [file mbo004173407sf7.tif]

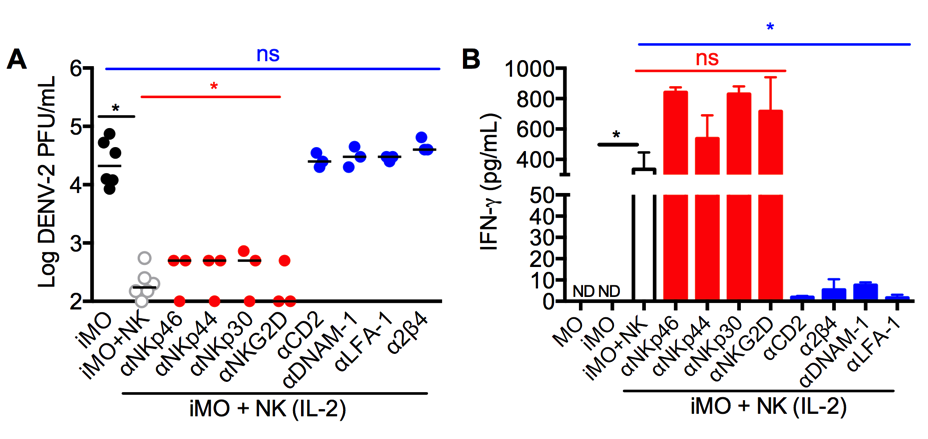

Supplement: FIG S8 [file mbo004173407sf8.tif]
